# The TyphiNET data visualisation dashboard: unlocking *Salmonella* Typhi genomics data to support public health

**DOI:** 10.1186/s13073-025-01470-4

**Published:** 2025-05-09

**Authors:** Zoe A. Dyson, Louise Cerdeira, Vandana Sharma, Megan E. Carey, Kathryn E. Holt, Global Typhoid Genomics Consortium, David M. Aanensen, Ali H. Abbas, Antoine Abou Fayad, Ayorinde O. Afolayan, Niyaz Ahmed, Irshad Ahmed, Afreenish Amir, Saadia Andleeb, Silvia Argimón, Abraham Aseffa, Philip M. Ashton, Mabel K. Aworh, Ashish R. Bavdekar, Marie A. Chattaway, Ka Lip Chew, John A. Crump, Thomas C. Darton, Paula L. Diaz, Christiane Dolecek, Nicholas A. Feasey, Andrew R. Greenhill, Madhu Gupta, Mochammad Hatta, Rene S. Hendriksen, Robert S. Heyderman, Odion O. Ikhimiukor, Aamer Ikram, Danielle J. Ingle, Arti Kapil, Jacqueline A. Keane, Karen H. Keddy, Robert A. Kingsley, Myron M. Levine, Calman A. MacLennan, Mailis Maes, Jaspreet Mahindroo, Tapfumanei Mashe, Masatomo Morita, Elli Mylona, Geetha Nagaraj, Satheesh Nair, Take K. Naseri, Elisabeth Njamkepo, Sophie Octavia, Iruka N. Okeke, Michael Owusu, Maria Pardos de la Gandara, Andrew J. Pollard, Sadia I. A. Rahman, Saikt Rahman, David A. Rasko, Elrashdy M. Redwan, Assaf Rokney, Priscilla Rupali, Jean Pierre Rutanga, Jivan Shakya, Senjuti Saha, Michael J. Sikorski, Anthony M. Smith, Kaitlin A. Tagg, Neelam Taneja, Dipesh Tamrakar, Paul Turner, James E. Ussher, Sandra Van Puyvelde, Koen Vandelannoote, François-Xavier Weill, Vanessa K. Wong, Jackie Wright

**Affiliations:** 1 Department of Infection Biology, Faculty of Infectious and Tropical Diseases, London School of Hygiene & Tropical Medicine, London, WC1E 7HT UK; 2 Department of Infectious Diseases, School of Translational Medicine, Monash University, Melbourne, VIC 3004 Australia; 3 Wellcome Sanger Institute, Wellcome Genome Campus, Hinxton, UK

**Keywords:** Typhoid fever, *Salmonella* Typhi, Antimicrobial Resistance, Whole Genome Sequencing, Genomics, Genetic epidemiology, Surveillance, Dashboard, Web application

## Abstract

**Background:**

*Salmonella enterica* subspecies *enterica* serovar Typhi (abbreviated as ‘Typhi’) is the bacterial agent of typhoid fever. Effective antimicrobial therapy reduces complications and mortality; however, antimicrobial resistance (AMR) is a major problem in many endemic countries. Prevention through vaccination is possible through recently-licensed typhoid conjugate vaccines (TCVs). National immunisation programs are currently being considered or deployed in several countries where AMR prevalence is known to be high, and the Gavi vaccine alliance has provided financial support for their introduction. Pathogen whole genome sequence data are a rich source of information on Typhi variants (genotypes or lineages), AMR prevalence, and mechanisms. However, this information is currently not readily accessible to non-genomics experts, including those driving vaccine implementation or empirical therapy guidance.

**Results:**

We developed TyphiNET (https://www.typhi.net), an interactive online dashboard for exploring Typhi genotype and AMR distributions derived from publicly available pathogen genome sequences. TyphiNET allows users to explore country-level summaries such as the frequency of pathogen lineages, temporal trends in resistance to clinically relevant antimicrobials, and the specific variants and mechanisms underlying emergent AMR trends. User-driven plots and session reports can be downloaded for ease of sharing. Importantly, TyphiNET is populated by high-quality genome data curated by the Global Typhoid Pathogen Genomics Consortium, analysed using the Pathogenwatch platform, and identified as coming from non-targeted sampling frames that are suitable for estimating AMR prevalence amongst Typhi infections (no personal data is included in the platform). As of February 2024, data from a total of *n* = 11,836 genomes from 101 countries are available in TyphiNET. We outline case studies illustrating how the dashboard can be used to explore these data and gain insights of relevance to both researchers and public health policy-makers.

**Conclusions:**

The TyphiNET dashboard provides an interactive platform for accessing genome-derived data on pathogen variant frequencies to inform typhoid control and intervention strategies. The platform is extensible in terms of both data and features, and provides a model for making complex bacterial genome-derived data accessible to a wide audience.

**Supplementary Information:**

The online version contains supplementary material available at 10.1186/s13073-025-01470-4.

## Background

*Salmonella enterica* subspecies *enterica* serovar Typhi (abbreviated as ‘Typhi’) is the bacterial agent of typhoid fever [1], a faeco-orally transmitted systemic bacterial infection, which sickens an estimated nine million people each year [2]. Most illnesses occur in low- to middle-income countries (LMIC) in settings with insufficient sanitation infrastructure, microbiologically unsafe water and food, and poor hygiene, where the disease burden is highest among children [3]. Effective antimicrobial therapy hastens the resolution of typhoid symptoms [4–6], reduces the risk of complications [7], and reduces mortality from ~ 10% to 1% [8, 9]. Formal diagnosis and susceptibility testing requires blood culture that has low sensitivity (< 60%) and is often limited or unavailable in high-burden settings [10, 11]. Therefore, therapy is often empiric, and guided by local antimicrobial resistance (AMR) patterns rather than direct testing. For example, the World Health Organization (WHO) ‘AWaRe (Access, Watch, Reserve) Antibiotic Book’ [12] recommends treating suspected typhoid with ciprofloxacin if the local prevalence of resistance is low, and oral azithromycin for uncomplicated disease or intravenous ceftriaxone for severe disease if local prevalence of ciprofloxacin resistance is high. The former first-line drugs ampicillin, chloramphenicol, and trimethoprim-sulfamethoxazole have not been recommended by the WHO for typhoid fever since the 1990 s, when multidrug resistance (MDR, defined as resistance to these three agents) became common [5, 13]. Extensively drug resistant (XDR) strains have been reported and these are MDR strains that are resistant to ciprofloxacin and ceftriaxone. Patients with uncomplicated disease due to XDR Typhi may be treated with azithromycin, and carbapenems are used for severe disease but are problematic due to cost and the need for intravenous administration.

While vaccines to prevent typhoid have been available for decades, they have not been widely implemented in endemic regions. The situation is changing now due to the prequalification of typhoid conjugate vaccines (TCVs) by the World Health Organization (WHO) in 2018. TCVs are safe and effective in children and in infants as young as six months of age [13]. TCVs are now eligible for Gavi support, providing a potentially affordable route for low-income countries to invest in typhoid prevention through national immunisation programs [14]. As AMR has an impact on the clinical outcomes of Typhi infections, it is not only the burden of typhoid fever but also the prevalence of AMR that needs to be considered when weighing the costs and benefits of disease prevention through vaccination [15]. Indeed, TCV was the first vaccine to be recommended by the WHO based partially on pathogen-specific AMR concerns. For example, XDR typhoid outbreaks in Pakistan and ciprofloxacin resistant (CipR) outbreaks in Zimbabwe prompted responsive TCV roll-out in affected areas, which were effective in reducing local disease incidence [16–21]. Such responses have since been followed by introduction of national immunisation programs, with Pakistan being the first country to introduce TCV into its routine immunisation schedule.

Country-level AMR prevalence data are important to inform both empiric treatment of typhoid fever, and make the case for investment in national immunisation programs. *Salmonella enterica* is included in the WHO Global Antimicrobial Resistance and Use Surveillance System (GLASS) [22], but is not currently disaggregated by serovar. Local data on Typhi AMR remain scarce in most LMICs, and are mostly gathered in the context of time-limited research studies, outbreak investigations, or from travellers returning to other countries that have routine surveillance [23]. While informative, these data are not collected consistently, are predominantly based on phenotypic testing, and methods and interpretive criteria can vary by country and over time, which complicates reporting and interpretation. Whole genome sequencing (WGS) data are increasingly adopted as the standard for strain characterisation of Typhi [24–26], and a hierarchical genotyping and nomenclature scheme (GenoTyphi) has been developed to aid the detection and tracking of lineage variants [27, 28]. The genetic determinants of AMR in Typhi are well understood [5, 24, 29], such that AMR phenotypes can also be predicted from WGS data, with a recent study demonstrating 99.9% concordance between AMR genotypes and phenotypes assessed in the English reference laboratory [26]. Consequently, WGS is now a standard method of characterisation in routine surveillance of typhoid in reference laboratories in many high-income countries [23, 26, 30, 31], as well as in research studies globally, making WGS data a rich source of information on Typhi pathogen diversity and local AMR patterns [24].

As AMR continues to evolve and spread, WGS-based pathogen surveillance has the potential to inform public health policies for typhoid such as empirical therapy guidelines [32]; water, sanitation, and hygiene (WASH) interventions that could impact pathogen transmission [33]; and national vaccine introduction decision making [34, 35]. However, at present, these data are not universally accessible to decision-makers at different levels of public health policy nor presented in a format that can inform on national prevalence of AMR. Typhi genome data are browsable in various public databases including the National Center for Biotechnology Information (NCBI) Pathogen Detection portal [36], Enterobase [37, 38], BIGSdb [39], and Pathogenwatch [29]. However, these databases (i) are designed for a user base with expertise in genomics, bioinformatics, and WGS analysis; (ii) include WGS data from heterogeneous sources, including many that are not suitable for surveillance of AMR prevalence (e.g., outbreak investigations [40], or studies that specifically sequence resistant strains to ascertain mechanisms [41]); and (iii) do not provide country-level summaries of key variables such as genotype and AMR prevalences since uncurated bundles of input data are not suitable for this.

Here, we present the TyphiNET genomic surveillance dashboard for typhoid (available at: https://www.typhi.net), which aims to make genome-derived data on Typhi genotypes and AMR accessible to a broad user-base. TyphiNET is powered by existing informatics solutions for Typhi genomic analysis (including the GenoTyphi framework and Pathogenwatch platform), and leverages contextual metadata collected and curated by the Global Typhoid Genomics Consortium (GTGC), to filter and analyse raw heterogeneous public genome data and extract meaningful AMR and genotype (variant) prevalences. Where sufficient input data are available, the dashboard also allows visualisation of temporal trends, and users can interrogate the association of specific AMR determinants with genotype backgrounds.

## Implementation

### Dashboard architecture

**Fig. 1:**
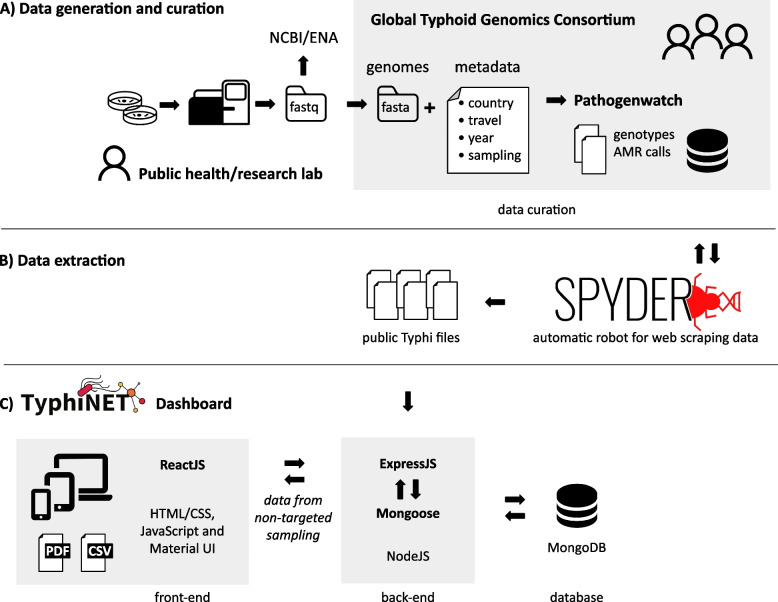
TyphiNET data curation and dashboard architecture. **A** The Global Typhoid Genomics Consortium (GTGC) aggregates and curates Typhi genome data and metadata, using Pathogenwatch as both an analysis platform (calling genotypes and AMR determinants from genome assemblies) and publicly accessible data store. Metadata that are not typically available in NCBI/ENA but are collected and curated by the GTGC include purpose of sampling (to tag datasets that are suitable for estimating AMR/genotype prevalence) and information on country-of-travel for travel-associated isolates (to identify country of origin). **B** A web-scraper is used to pull the latest versions of genotypes, AMR determinants, and metadata files from GTGC-curated Typhi collections in Pathogenwatch, which are used to populate the TyphiNET database. **C** The TyphiNET dashboard is implemented as a MERN (MongoDB, Express, React, Node) stack JavaScript application as illustrated. Genome data are filtered to exclude low-quality genome sequences, and data sets whose sampling frames make them unsuitable for AMR surveillance (such as those targeted towards sequencing of resistant strains only, or outbreak investigations), before calculating national/annual prevalences of AMR and genotypes to display in interactive plots. ReactJS is used to provide user interface layouts suitable for viewing the interactive plots on a range of devices (computer, tablet, phone). Users can also download static images of current plot displays (PNG), static reports with all current plots (PDF format), or a copy of the TyphiNET database (CSV format)

TyphiNET was developed as an open-source MERN (MongoDB, Express, React, Node) stack JavaScript application (Fig. 1). Front-end visualisations are implemented via ReactJS libraries, while back-end operations are implemented using ExpressJS and NodeJS. Genome-derived AMR and genotype data, and curated contextual metadata (see below), are retrieved from the collaborating Pathogenwatch platform [29] via an automatic robot for web scraping, dubbed Spyder v2.0 [42], and injected into the TyphiNET MongoDB Atlas via the back-end. The web application is deployed using the Heroku platform. All code is freely available under a GNU-GPL 3.0 licence via GitHub, the version described in this manuscript is v1.6, DOI: 10.5281/zenodo.14887041, which includes data updated on February 17 th 2024 [43]. We recommend viewing TyphiNET using Google Chrome v131.0.6778.140 or greater for best performance.

### Data curation and processing

In 2021 the GTGC was established to provide a community that could routinely aggregate Typhi WGS data to facilitate monitoring the emergence and spread of AMR and inform targeted public health action against typhoid fever. Soon after its establishment, the GTGC aggregated all publicly available Typhi sequence data and metadata generated to date. Data owners identified from professional networks, literature searches, and Nucleotide Sequence Database Collaboration (INSDC) databases (i.e., NCBI, EMBL-EBI, DDBJ) were contacted to provide corresponding source information captured using a standardised metadata template (detailed below). Subsequently, WGS data generated by research or public health laboratories and deposited in INSDC databases are assembled and quality filtered by the GTGC [24], and uploaded to Pathogenwatch [29] for analysis (Fig. 1). Typhi Pathogenwatch screens assemblies for known determinants of AMR [29] and carries out lineage assignment according to the GenoTyphi genotyping framework [28, 44]. Full details on GTGC data collection approaches are provided in Carey et al. 2023 [24].

The GTGC curates contextual metadata (i.e., source data) associated with each Typhi genome sequence, to enhance the re-usability of the WGS data. This is done via requesting data generators (most of whom are GTGC members) to complete a standardised metadata template [24]. Key fields in the GTGC metadata template that are not commonly or consistently included in metadata submitted to INSDC or supporting publications, but which are important for re-using genome data for AMR surveillance, are (i) travel information (Travel Associated: yes or no, Country of Travel); (ii) purpose of sampling (Targeted: Cluster Investigation, AMR investigation, Other; or Non Targeted: Reference lab, Surveillance Study, Routine diagnostics, Other); (iii) identifying repeat isolates; and (iv) data to confirm case status (Host Health State: Symptomatic or Asymptomatic Carrier; Source: Blood, Stool, Environment or Food). Repeat isolates, defined as those that represent the same occurrence of typhoid infection, are excluded such that only a single 'primary' isolate (either the first, or the best quality genome, for each unique case) are included in the GTGC data set [24]. ‘Country of origin’ is defined as the country where the pathogen was isolated, or for travel-associated infections, the country recorded as the presumed country of infection based on travel history [23, 45–47]. The GTGC-curated metadata (including ‘Country of Origin’, and with repeat isolates removed) is processed and uploaded to Pathogenwatch along with the sequence data, to generate curated ‘Collections’ in Pathogenwatch (one for each source study or public health lab). The Spyder API [42] is then used to retrieve data files from Pathogenwatch for the GTGC-curated collections, for injection into the TyphiNET database.

In the TyphiNET dashboard, genomes are further filtered to include only those recorded as coming from non-targeted sampling frames (Reference lab, Surveillance Study, Routine diagnostics, Other). Those from targeted sampling frames (Cluster investigation, AMR focused, Other) or unspecified sampling frames are excluded from dashboard analyses and visualisations to prevent biases in prevalence estimates (although they are included in the database download, for completeness and for use in other analyses outside the dashboard). The TyphiNET dashboard also filters out cases recorded as asymptomatic carriers (*n* = 119) or coming from gallbladder (*n* = 1) or environmental (*n* = 14) samples; the rest are assumed to represent acute illness (including *n* = 9,039 recorded explicitly as blood isolates and/or symptomatic typhoid assumed to be acute).

### Statistical and design considerations

Typhoid intervention strategies such as immunisation programs and changes in empirical therapy are typically implemented at a country level, by national immunisation technical advisory groups (NITAGs) and ministries of health [48]. The TyphiNET dashboard therefore focuses on country as the geographical unit, to report national annual prevalences of genotypes and AMR aggregated from all available data sources for that country. Prevalence estimates are simple proportions (expressed as percentages), calculated from the data in the curated TyphiNET database (i.e. non-repeat assumed-acute cases from non-targeted sampling frames, as outlined above). Where multiple data sources are available for a given country, the prevalence is a simple weighted pooled estimate calculated by summing the numerators and denominators across all available data for the given country and the selected time period. The minimum sample size to report a national annual prevalence is N ≥ 10 (equivalent to current WHO GLASS reports [22], which require data from 10 individuals in order to report a national prevalence rate). The minimum sample size to report a national prevalence on the map is N ≥ 20. The number of samples, and number and scope of available data sources, varies substantially by country; however, previous robustness analyses reported by the GTGC show that, for countries with multiple data sources (e.g., burden studies in different cities, and returning traveller data collected in other countries), the per-data-set prevalence estimates are largely concordant with the pooled country-wide estimates [24].

Unless otherwise stated, statistical analyses presented in case studies were conducted using base R (v4.4.1).

### AMR definitions

Binary variables representing predicted resistance phenotypes are calculated from the AMR genes and Single Nucleotide Polymorphisms (SNPs) reported by Pathogenwatch [29], as summarised in Table 1. Note that there are limited data to assess the clinical significance of *acrB* mutations on treatment response to azithromycin, for simplicity we refer to isolates carrying these as azithromycin resistant [41].

**Table 1:** Definitions used to calculate AMR variables. Genetic determinants reported by Pathogenwatch are used to calculate binary resistance prediction variables in TyphiNET

Predicted Resistance	Genetic determinants
Ampicillin/amoxicillin	*bla* gene
Azithromycin	*acrB*-R717Q or *acrB*-R717L mutation
Ceftriaxone	≥ 1 ESBL gene^a^
Chloramphenicol	*catA1* or *cml1* gene
Ciprofloxacin non-susceptible (CipNS) (MIC, > 0.06 mg/L) [49–51]	≥ 1 QRDR^b^ mutation, and/or ≥ 1 *qnr* gene
Ciprofloxacin resistant (CipR) (MIC, > 0.5 mg/L) [49–51]	≥ 3 QRDR^b^ mutations, or ≥ 1 QRDR^b^ plus ≥ 1 *qnr* gene^c^
Sulphonamides	≥ 1 *sul* gene
Tetracyclines	≥ 1 *tetA* gene
Trimethoprim	≥ 1 *dfrA* gene
Trimethoprim-sulfamethoxazole	≥ 1 *sul* gene Plus ≥ 1 *dfrA* gene
Multidrug resistant (MDR; resistance to chloramphenicol plus ampicillin plus trimethoprim-sulfamethoxazole)	*catA1* or *cml1* gene Plus *bla* gene Plus ≥ 1 *sul* gene Plus ≥ 1 *dfrA* gene
Extensively drug resistant (XDR; MDR plus CipR plus ceftriaxone resistant)	As for MDR Plus CipR Plus ≥ 1 ESBL gene
Pansusceptible (No AMR determinants detected)	None

^a^ESBL = extended spectrum beta-lactamase gene (those currently detected in the Typhi genomes are *bla*_CTX-M-12_*, bla*_CTX-M-15_*, bla*_CTX-M-55_*, bla*_OXA-134_*, bla*_SHV-12_)

^b^QRDR = quinolone resistance determining region mutations tracked by Pathogenwatch [29] (these are currently *gyrA* S83 and D87, *gyrB* S464, *parC* S80 and E84)

### Global map view

The first visualisation panel summarises country-level data on a world map developed using the react-simple-maps JavaScript library [52]. Users can choose to colour countries in the map by: (i) national prevalences of clinically relevant AMR profiles including MDR, XDR, CipNS, CipR, AziR, pansusceptible (see Table 1), (ii) national prevalence of genotypes (lineage variants), including the dominant genotype per country and the prevalence of genotype 4.3.1; or (iii) number of samples available. By default, the map view shows values enumerated from all Typhi isolates, sampled both locally in-country and travel-associated cases captured in other countries. Users can choose to filter to either local or travel data only (via a toggle button) and/or to filter on a specified time window (by selecting start and end years). These filters apply to all dashboard plots, not just the map.

AMR prevalences per country are indicated visually on the world map, using increasing colour intensity to signal categorical prevalence ranges of escalating concern with respect to use of the drug for empiric therapy: (i) 0, no resistance detected; (ii) > 0 and ≤ 2%, resistance present but rare; (iii) > 2 and ≤ 10%, resistance uncommon; (iv) > 10% and ≤ 50%, resistance common; (v) > 50%, established resistance. The ‘sensitive to all drugs’ plot (selected via the ‘map view’ dropdown menu) is coloured differently, to draw attention to countries with low prevalence of pansusceptible strains and thus where choice of antimicrobial is most important: (i) < 10% pansusceptible; (ii) > 10 and ≤ 20%; (iii) > 20 and ≤ 50%; (iv) > 50 and ≤ 90%; and (v) > 90%. Prevalence estimates are visualised where ≥ 20 sequences are available for a given country and timeframe, otherwise “Insufficient data” is shown (light grey; Additional File 1 Fig. S1a).

Users can interact with the map by hovering the mouse cursor over a country, to view a tool-tip displaying the name of the country and the number (N) and percentage of genomes from that country that are resistant (or pansusceptible or H58/4.3.1, depending on the ‘map view’ variable selected); numbers shown always reflect the current choice of local/travel and temporal filters. Selecting ‘No. Samples’ as the ‘map view’ variable to plot colours the map according to number of samples available using the current filters; in this view, hovering over a country reveals a tool-tip displaying the name of the country, number of samples and genotypes, and prevalence of H58/4.3.1 and each of the AMR categories.

### Detailed plots (for country-level data)

The second visualisation panel includes four additional data plots designed to highlight annual trends in genotype and AMR prevalences, AMR prevalence within genotype, and molecular mechanisms underlying AMR. Upon loading the dashboard, these plots are populated by the full dataset (i.e., all countries), however, they were designed mainly for the purpose of showing detail for a single country of interest. Users can select a country by clicking it on the map, or selecting its name from the ‘select country’ dropdown menu below the map. The data plots are then populated by filtering the database to include only samples from the selected country of origin (along with applying any local/travel and time filters selected in the map panel).

The ‘Drug resistance trends’ plot (Additional file 1: Fig. S1c) shows annual pooled global prevalence of genomically-predicted resistance to the drugs listed in Table 1 (as well as prevalence of genomes identified as pansusceptible). By default, trend lines are plotted for the most currently relevant AMR categories (MDR, XDR, CipR, CipNS, AziR, CefR, Trimethoprim-sulfamethoxazole resistant, pansusceptible), but individual variables can be hidden or displayed via the dropdown menu ‘Select drugs/classes to display’. For the remaining three plots (Additional File 1: Fig. S1b,d,e), users can visualise data as either counts or percentages using dropdown menus. For the ‘Resistance frequencies within genotypes’ and ‘Resistance determinants within genotypes’ plots, percentages are shown by default to highlight the most resistant pathogen variants. By default, the ‘Resistance frequencies within genotypes’ plot shows the top five most-resistant genotypes in the currently-selected country, but other genotypes can be selected via ‘data view’ dropdown menu. The ‘Resistance determinants within genotypes’ plot shows the genetic determinants underlying resistance in up to the 10 most-resistant genotypes for the currently-selected filters and a selected drug category. The default view is of determinants conferring non-susceptibility to ciprofloxacin, as this is the most relevant to empiric treatment choice, however other drugs can be selected from the ‘drug class’ dropdown menu. For the ‘Genotype distributions’ plot, counts are shown by default to mimic epidemic curves used in epidemiological investigations for case counts. As for the map views, hovering the mouse cursor over these plots reveals a tool-tip displaying the raw data count (N) and prevalence (%) underlying each data point.

### Static outputs

User-generated visualisations can be downloaded individually as portable network graphics (PNG) files, and a report of all current data visualisations can be downloaded as a portable documents format (PDF) file (Additional file 1: Fig. S1f; example output in Additional file 2). If a country is selected, the report includes a list of publications (PubMed IDs) for genomes included in the current view, to facilitate proper citation and provenance-tracking of constituent datasets (otherwise the report refers readers to download the database for this information). A plain text line list (comma separated values, CSV) of the full TyphiNET database is also available for download (example output in Additional file 3). This file contains the GTGC-curated sample metadata (including country of origin, year of isolation, purpose of sampling, travel association; provenance information for individual genomes such as originating lab, primary publication PubMed ID, sequence data accession numbers), as well as genome-derived AMR determinants and pathogen genotypes assigned by Pathogenwatch. This file is intended to facilitate provenance-tracking of individual genome sequences, and to allow expert users to further explore the data using other tools.

## Results and discussion

**Fig. 2:**
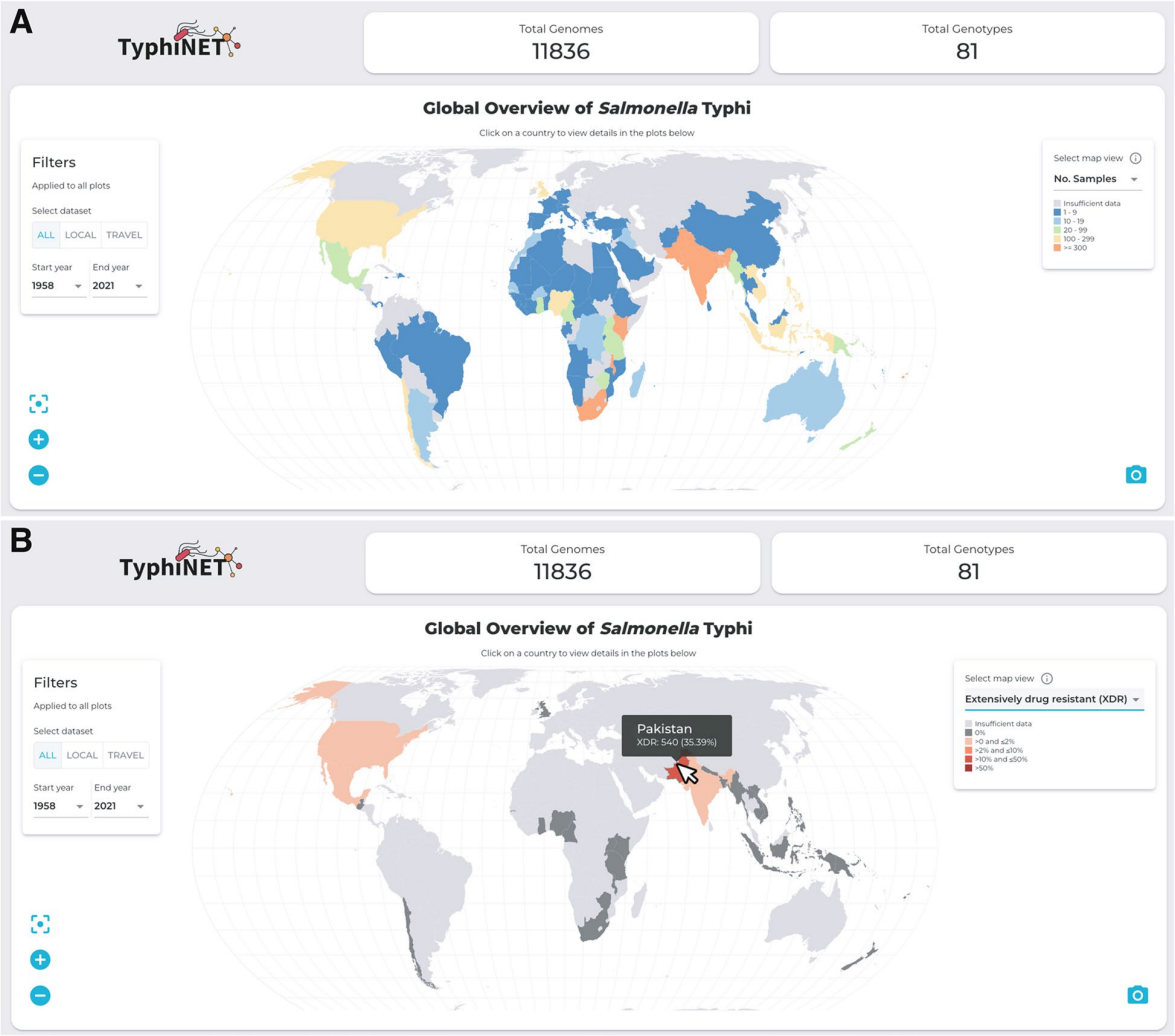
Global views of data gaps and AMR prevalence. **A** Total sample counts per country. Top panels indicate the number of sequences and genotypes present in the TyphiNET dashboard as of February 2024. Left panel indicates controls for filtering the data visualised by data source (all data, locally collected cases, or travel-associated cases) and time period (by providing start and end years for the period). Countries on the map are coloured by the total number of samples as per the inset legend (top right of map). **B** National frequencies of XDR. Countries on the map are coloured by XDR frequency as per the inset legend (top right of map). Data are shown where there are ≥ 20 sequences available for the country of interest. Tool tip indicates summary statistics for Pakistan upon mouse over

Version 1.6 of the TyphiNET database (February 2024) included data derived from 12,671 genomes curated by the GTGC, including those described in the initial GTGC publication [24] and more recent published datasets from Kenya [53] and Fiji [54]. The TyphiNET dashboard displays data from genomes from assumed-acute typhoid cases from non-targeted sampling frames (see Implementation), resulting in a total of *n* = 11,836 Typhi in version v1.6. The filtered database contains data from 101 countries, however as the map view of sample counts shows (Fig. 2a), most countries are represented by very few samples. Prevalence estimates are calculated only for countries represented by ≥ 20 sequences, currently *n* = 30 countries (see e.g., map of XDR prevalence, Fig. 2b). The database currently includes samples from 1958–2021, with the majority from 2010 onwards (*n* = 10,382, 87.7%). High-burden countries in South Asia are well represented with ≥ 1,300 genomes each (Bangladesh, *n* = 1,664; India, *n* = 2,327; Nepal, *n* = 1,300; Pakistan, n = 1,526), including both local data from large-scale disease-burden studies [55, 56] and travel-associated infection isolates sequenced in other countries (Bangladesh, 14.1%; India, 45.2%; Nepal, 1.8%; Pakistan, 39.6%) [23, 26]. African countries are currently represented by low numbers of genomes, with ten countries exceeding 20 genomes (Malawi *n* = 568; Kenya *n* = 824; Nigeria *n* = 170; Ghana *n* = 69; Rwanda *n* = 52; South Africa *n* = 312; Uganda *n* = 36; Tanzania *n* = 33; Cameroon *n* = 27; Gambia *n* = 24). This highlights the need for culture-based surveillance studies in typhoid endemic countries in Africa [57]. Notably TyphiNET and the GTGC provide a mechanism for continued updating of the database as new genomes are released from such efforts, as well as from travel-associated infections captured in other countries. For example, Nigeria is currently represented by *n* = 28 travel-associated infections and *n* = 142 local infections, which reflect the same general trends in terms of dominance by genotypes 3.1.1 and 2.3.1, with higher prevalence of MDR in 3.1.1 highlighted by both data sources (Additional file 1: Fig. S2).

We conducted an informal assessment of dashboard useability by asking untrained users from different geographies to explore the dashboard and use it to answer 10 multiple-choice questions. Questions were designed to assess whether users could successfully interact with the dashboard in order to find the answers to specific questions regarding the prevalence, trends, and determinants of typhoid fever resistance in specific countries and time periods (Table 2). Our goal was to assess the ability of users who are generally familiar with typhoid and AMR to find the specific information they need, rather than to assess comprehension or understanding of typhoid and AMR. Therefore, the audience for the quiz was members of the Global Typhoid Genomics Consortium and their colleagues at academic and public health institutions, and we did not track the professional background or expertise of individual respondents, nor did we provide any training or background to typhoid fever or the concepts employed in the dashboard. The results (from *n* = 42 respondents) suggest the dashboard is sufficiently intuitive for users who are familiar with the concepts, but not familiar with the dashboard interface, to find the correct answers to these types of questions (> 90% correct responses to 8/10 questions, see Table 2).

**Table 2:** Informal assessment of dashboard useability

Question	Answers (*Correct)	Correct responses N (%)
In which country is extensively drug-resistant (XDR) *S.* Typhi most prevalent (across all years)?	India Mexico Pakistan * United States	41 (98%)
How many total genomes are available from Nepal since 2010?	1100 1274 * 1484 1660	39 (93%)
How many of the genomes available from Nepal since 2010 are from travel data?	20 * 45 75 103	41 (98%)
What percentage of S. Typhi from Kenya isolated between 2010 and 2020 were multidrug resistant (MDR)?	23.45% 43.29% 78.41% * 100%	39 (93%)
What is the overall trend in Ciprofloxacin non-susceptibility (CipNS) in Kenya from 2010 to 2020?	Increasing * Decreasing No change	41 (98%)
Which genotypes had the highest rates of Ciprofloxacin non-susceptibility (CipNS) in Kenya from 2010–2020? (two answers)	4.3.1.1.EA1 4.3.1.2.EA2 * 4.3.1.2.EA3 * 0.0.1 4.3.1.1.EA2 4.3.1.1.EA3	34 (81%) selected ONE or BOTH correct genotypes
What was the most common genotype in Nigeria in 2013?	2.3.1 2.3.2 3.1.1 * 4.1	41 (98%)
^a^What is the overall trend in prevalence of this genotype in Nigeria between 2010 and 2020?	Increasing Decreasing Stable	11 (26%) 17 (16%) 24 (57%)
What is the prevalence of trimethoprim resistance in this genotype in Nigeria between 2010 and 2020?	45% 78% 89% * 100%	32 (76%)
Which resistance gene is responsible for the majority of trimethoprim resistance in this genotype in Nigeria between 2010 and 2020?	dfrA14 + sul2 dfrA15 * dfrA1 + sul1 dfrA1 + sul1 + sul2	40 (95%)

^a^This question has no objective answer, as the prevalence of genotype 3.1.1 in Nigeria moves up and down between 2010–2020

In the following sections, we present three case studies that highlight the utility of the TyphiNET dashboard as a tool for understanding national AMR trends and the underlying mechanisms and pathogen genotypes. The case studies were developed to illustrate previously documented shifts in local Typhi populations in African and Asian countries, each associated with different types of changes in AMR patterns (emergence or decline of resistance, and lineage replacement) that have implications for local typhoid control.

### Case study 1 : emergence and clonal expansion of XDR typhoid in Pakistan

**Fig. 3:**
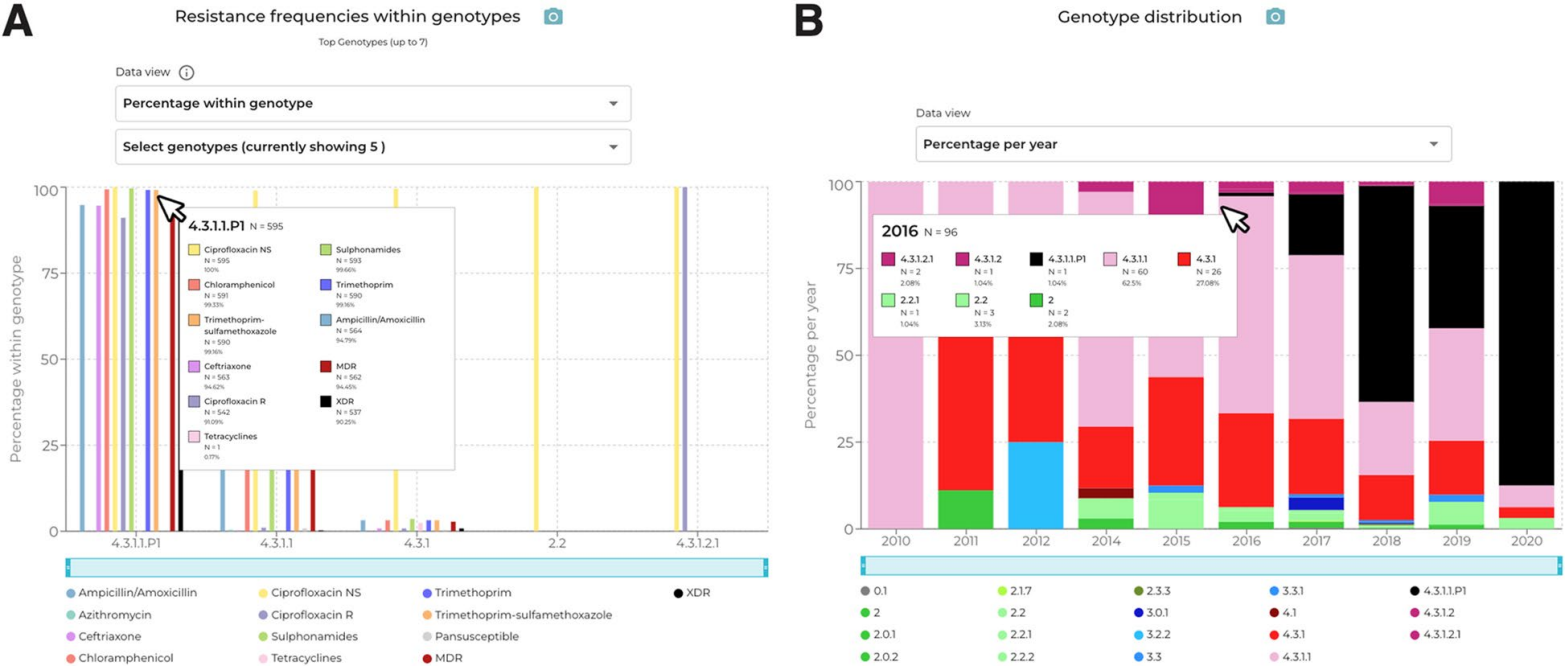
Exploring the emergence of XDR Typhi in Pakistan with the TyphiNET dashboard. **A** ‘Resistance frequencies within genotypes’ plot shows frequencies of resistance to different drug classes, within common genotypes circulating in Pakistan. Bars are coloured according to the inset legend. **B** ‘Annual genotype distribution’ plot shows the frequencies of pathogen genotypes circulating in Pakistan per year. Genotypes are coloured as per the inset legend

Selecting ‘Extensively drug resistant’ from the dropdown menu in the map, it is clear that Pakistan has many XDR cases (Fig. 2b). Toggling the ‘Local’ and ‘Travel’ filters shows that this high prevalence is evident in data from both sources. Clicking on Pakistan in the world map allows exploration of the genotypes, resistance mechanisms and annual trends underlying the high prevalence of XDR in the country. With the filter set to ‘All’ (i.e., including local and travel data) and year range from 2010 to 2021, the ‘Resistance trends’ plot shows there is sufficient data (N ≥ 10) per year from 2014–2020 to calculate annual prevalence values. The plot shows the first emergence of XDR in Pakistan in 2016 (Additional file 1: Fig. S3a), against a background of persistently high CipNS prevalence (> 96% throughout 2014–2020) and MDR (~ 60% in 2014–2017, rising to 84% in 2020; p = 0.02 two proportions z-test). The ‘Resistance frequencies within genotypes’ plot shows MDR focused in the 4.3.1.1 genotype background, with CefR, CipR and XDR (ie the combination of MDR + CefR + CipR) localised in the derived genotype 4.3.1.1.P1 (Fig. 3a, Additional file 1: Fig. S3b). The ‘Genotype distribution’ plot, with data view set to ‘Percentage per year’, shows the first appearance of genotype 4.3.1.1.P1 in 2016 (Fig. 3b, Additional file 1: Fig. S3c). Mousing over the year 2016 brings up the tooltip, which clarifies that there is a single genome of 4.3.1.1.P1 amongst total *N* = 96 for the year. The plot shows prevalence of 4.3.1.1.P1 increased in subsequent years, reaching 87.5% in 2020, with the minor genotypes reducing in prevalence continuing to persist.

The ‘Resistance determinants within genotypes’ plot shows the genetic basis for XDR in 4.3.1.1.P1. The default view shows fluoroquinolone resistance determinants, and highlights that CipR in the genotype 4.3.1.1.P1 is due to the combination of a single QRDR mutation and a *qnrS* gene (Additional file 1: Fig. S3 d). Most other genotypes also have a single QRDR mutation (resulting in CipNS, shown in yellow), indicating that high-level resistance could emerge in any of these strain backgrounds through additional *gyrA*/*parC* mutations [58, 59] or plasmid-mediated acquisition of *qnr* genes [41, 60, 61]. Interestingly, the plot also highlights the presence of a variant with three QRDR mutations (resulting in CipR, shown in red), genotype 4.3.1.2.1, which emerged in India some decades ago [24, 62] (the genotype timeline shows this was detected in Pakistan between 2016–2019, in parallel with XDR 4.3.1.1.P1). Selecting ‘Ceftriaxone’ from the dropdown menu shows resistance in 4.3.1.1.P1 is mainly due to *bla*_CTX-M-15_ (Additional file 1: Fig. S3e); the same gene is found in a single isolate of 4.3.1.1 and two of 4.3.1. Selecting ‘Trimethoprim-sulfamethoxazole’ from the dropdown menu shows resistance in 4.3.1.1.P1 is mostly due to *dfrA7* plus *sul1* and *sul2*, and that this combination is also common in the parent genotype 4.3.1.1 (Additional file 1: Fig. S3f), consistent with emergence of XDR by acquisition of *qnrS* + *bla*_CTX-M-15_ within the locally circulating MDR + QRDR 4.3.1.1 variant.

The TyphiNET dashboard conveys several key points about XDR typhoid, which reflect the emerging picture of the problem captured in the wider literature [40, 56, 63–65]. These include (i) time of emergence of XDR typhoid; (ii) that XDR cases are due to emergence and dissemination of a single variant, genotype 4.3.1.1.P1; (iii) the underlying mechanisms of resistance; (iv) that this genotype shares with its parent, 4.3.1.1, the MDR genes and single QRDR mutation but has acquired *qnrS* and *bla*_CTX-M-15_ to become XDR; (v) that the XDR 4.3.1.1.P1 has not established locally transmitting populations outside Pakistan, at least not by 2020 (this is still true as of April 2024, however more recent data are sparse due to a decline in travel and prioritisation of SARS-CoV- 2 sequencing during the COVID19 pandemic). Intercontinental transmission of XDR typhoid associated with travel has been reported in several countries [23, 30, 46, 66–69], however, to date only a single report of a localised outbreak has occurred outside Pakistan [70]. TyphiNET will provide a means of monitoring the emergence of new XDR strains, as well as the persistence of XDR Typhi 4.3.1.1.P1 within Pakistan and its eventual spread to other settings which may motivate more widespread TCV use. The latter is of critical importance as recent surveillance data have shown that ancestral populations of genotype 4.3.1.1 circulating in Pakistan have acquired *acrB* mutations conferring azithromycin resistance [71] (as can be seen by selecting ‘Azithromycin’ in the ‘Resistance determinants within genotypes’ plot), suggesting that 4.3.1.1.P1 may also be able to tolerate these mutations.

### Case study 2: decline of MDR and emergence of azithromycin resistance in Bangladesh

**Fig. 4:**
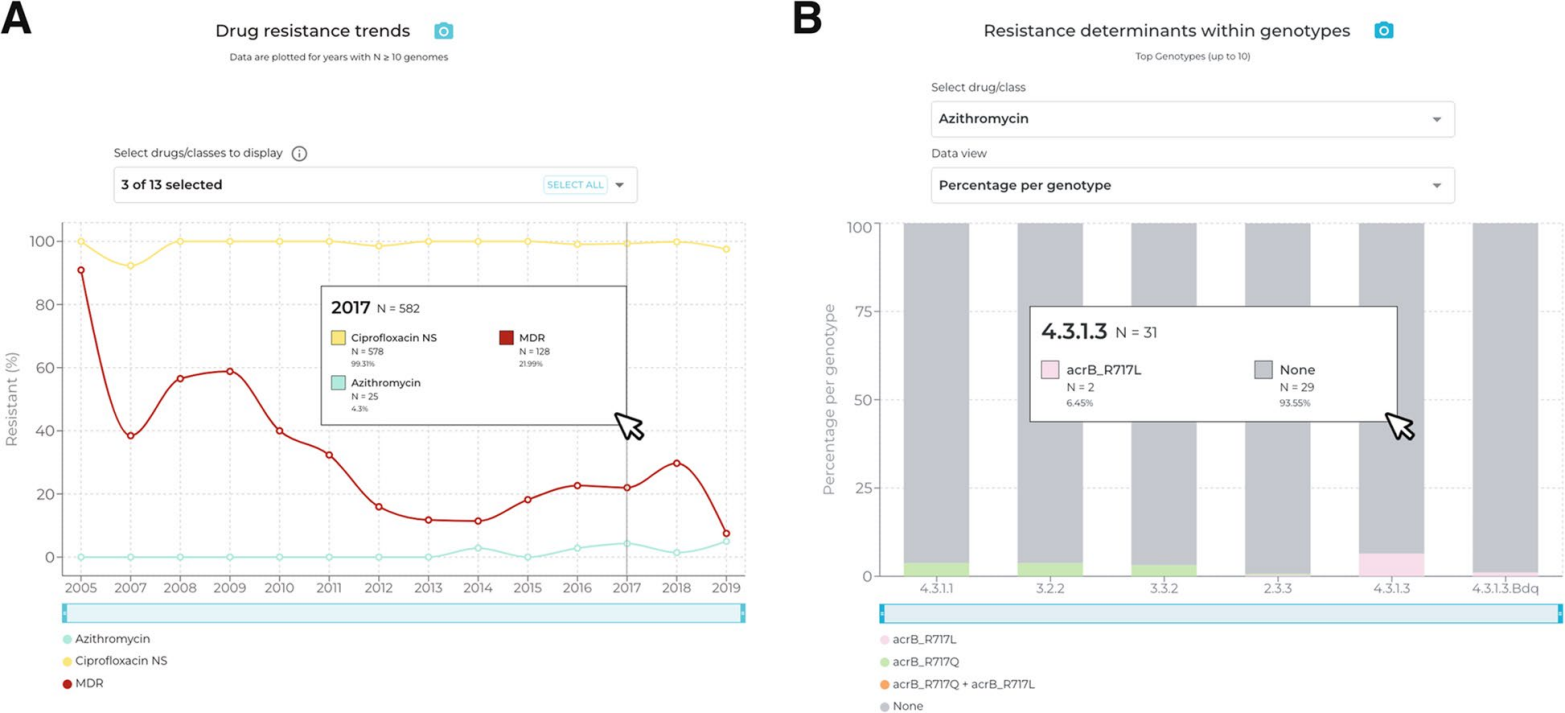
Azithromycin resistance emergence in Bangladesh is associated with different mutations in AcrB, arising in at least six different genotype backgrounds. **A** Resistance trends plot showing that ciprofloxacin non-susceptibility has remained near-universal since 2005, while MDR declined. Azithromycin emerged circa 2014, reaching 4–5% in 2017–2019. **B** Resistance determinants plot shows that two different types of azithromycin resistance mutations were detected (AcrB-R717L and AcrB-R717Q), in six different genotypes

Selecting ‘Multidrug resistant’ from the dropdown menu in the map, it can be seen that MDR infections are distributed throughout parts of sub-Saharan Africa, South-eastern Asia and South Asia (Additional file 1: Fig. S4a). Toggling the ‘Local’ and ‘Travel’ filters reveal a high prevalence of MDR cases in Bangladesh from both data sources. Clicking on Bangladesh in the world map, or using the ‘select country’ dropdown menu, allows exploration of country-level trends over time. With the filter set to ‘All’ (i.e., including local and travel data) and year range from 2005 to 2021, the ‘Resistance trends’ plot shows there are sufficient data (N ≥ 10 per year) to calculate annual AMR prevalences from 2005–2019 (with the exception of 2006) (Fig. 4a, Additional file 1: Fig. S4b). Mousing over the year 2005 reveals MDR (deep red line) was common in 2005 (91%), declining in subsequent years to 8–30% between 2013 and 2019 (*p* = 9.5 × 10^–9^ two proportions z-test). At the same time, CipNS (yellow line) remained high (> 92%) throughout 2005–2019 (Additional file 1: Fig. S4b). The ‘Genotype distribution’ plot, with data view set to ‘Percentage per year’ shows that 4.3.1 genotypes, including sublineages 4.3.1.1, 4.3.1.2, and 4.3.1.3, have also been gradually declining over the same time period (Additional file 1: Fig. S4c). Mousing over the year 2005 demonstrates that 91% (*n* = 10/11) were 4.3.1 genotypes (pink coloured bars), which declined to 20% (*n* = 8/40) in 2019 (*p* = 6.6 × 10^–6^ two proportions z-test). Over the same time period, genotype 3.3.2 (mid-blue coloured bars) persisted at low prevalence, and genotypes 2.0.1 and 2.3.3 (green bars) emerged and proliferated. Viewing the ‘Resistance frequencies within genotypes’ plot with default settings (data view as ‘Percentage within genotype’) demonstrates that MDR was only present in genotype 4.3.1.1 (72%), whereas CipNS was prevalent among all genotypes displayed (Additional file 1: Fig. S4 d). Selecting the local Bangladesh genotype ‘4.3.1.3.Bdq’ (Additional file 1: Fig. S4 d) from the dropdown menu, one can see this variant has a distinct AMR profile with resistance to ciprofloxacin, ampicillin, sulfonamides and tetracycline, but susceptibility to the older drugs chloramphenicol and trimethoprim as well as ceftriaxone. The ‘Resistance determinants within genotypes’ plot, when viewed with default settings (‘Select drug class’ set to ‘Ciprofloxacin’ and ‘Data view’ set to ‘Percentage per genotype’) reveals that most genotypes harbour a single QRDR mutation (yellow, resulting in CipNS), except for genotype 4.3.1.3.Bdq genomes which have acquired both a QRDR mutation and a *qnrS* gene (purple, resulting in CipR; Additional file 1: Fig. S4e). Despite being CipR, 4.3.1.3.Bdq strains do not appear to have replaced other co-circulating CipNS strains, with both 4.3.1.3.Bdq and CipR remaining at frequencies of < 18% between 2005–2019 (Additional file 1: Fig. S4c). It is tempting to speculate that this may be related in some way to the variant’s susceptibility to once commonly prescribed drugs chloramphenicol and trimethoprim, although it could also be due to a fitness cost associated with the IncFIB(K) plasmid it carries [60, 61].

The ‘Drug resistance trends’ plot also demonstrates the emergence of azithromycin resistance from 2014 onwards (Fig. 4a, Additional file 1: Fig. S4b; ≤ 5% of isolates per year up until 2019) in Bangladesh. The ‘Resistance frequencies within genotypes plot’, with default settings, shows that azithromycin resistance does not appear to be associated with any specific genotype and is occurring in multiple genotype backgrounds (Fig. 4b, Additional file 1: Fig. S4f). The molecular mechanisms driving azithromycin resistance among different pathogen genotypes can be viewed in the ‘Resistance determinants within genotypes’ plot by using the ‘Select drug class’ dropdown menu to select ‘Azithromycin’. This reveals the mechanism is a mix of non-synonymous mutations at *acrB* codon 717, with *acrB*-R717Q found in four genotypes and *acrB*-R717L in three genotypes (Fig. 4b, Additional file 1: Fig. S4f), including a single example of each mutation in genotype 2.3.3. Examining the global distribution of azithromycin resistance on the map, by selecting ‘Azithromycin resistant’ from the ‘map view’ dropdown menu, highlights that the burden of resistant strains, at present, is low and largely concentrated among South Asian countries (Additional file 1: Fig. S5).

TyphiNET captures several key aspects of the population dynamics and evolution of AMR among Typhi populations in Bangladesh observed across multiple surveillance studies [41, 55, 56, 60, 61, 72]. These include (i) the decline of MDR over the last two decades, coinciding with a decline in 4.3.1 genotypes; (ii) a sustained high frequency of CipNS cases driven by a diverse range of pathogen genotypes, mostly carrying a single QRDR mutation; (iii) the continued presence of CipR genotype 4.3.1.3.Bdq; and (iv) the timeframe and molecular mechanisms driving the emergence of azithromycin non-susceptibility due to mutations in *acrB* across multiple pathogen genotypes [41, 55, 56, 72]. Fortunately, there does not yet appear to be local establishment or geographical spread of any specific azithromycin-resistant variants (Additional file 1: Fig. S5), however the aggregation of genomic data from multiple sources in the TyphiNET dashboard could facilitate identifying and tracking the emergence of such clones in future.

### Case study 3: replacement of susceptible genotypes in Malawi with MDR genotype 4.3.1.1

Choosing ‘Multidrug resistant’ from the map dropdown menu shows a few countries with MDR prevalence exceeding 50% (dark red), including Malawi. Setting the time window to 2010 onwards and hovering the mouse cursor over Malawi shows the estimated MDR prevalence for this period is 93% (Additional file 1: Fig. S6a). With the country set to Malawi, the travel filter set to ‘All’ (i.e., including local and travel data) and year range from 2010 to 2019, the ‘Drug resistance trends’ plot shows that MDR prevalence (deep red line; Additional file 1: Fig. S6b) has risen steeply over this decade (this line can be seen more clearly by de-selecting ‘Trimethoprim-sulfamethoxazole’ from the ‘Drugs view’ menu). The proportion amongst the 19 isolates sequenced in 2010 was relatively low (21%), but reached 96% (*n* = 25/26) in 2012 and has been persistently high (> 95%) since then (*p* = 1.0 × 10^–7^ two proportions z-test). The ‘Genotype distribution’ plot shows that over the same period there was clonal replacement, with the diverse genotypes that were present in 2010 being replaced by genotype 4.3.1.1.EA1 (light pink bars; Additional file 1: Fig. S6c). Hovering the mouse over the graph shows that 4.3.1.1.EA1 prevalence rose from 21% in 2010 to 96% in 2012, and has remained above 95% ever since (*p* = 1.0 × 10–7 two proportions z-test; Additional file 1: Fig. S6c). The ‘Resistance frequencies within genotypes’ plot demonstrates that resistance to first line drugs is almost entirely associated with genotype 4.3.1.1.EA1 (Additional file 1: Fig. S6 d).

**Fig. 5:**
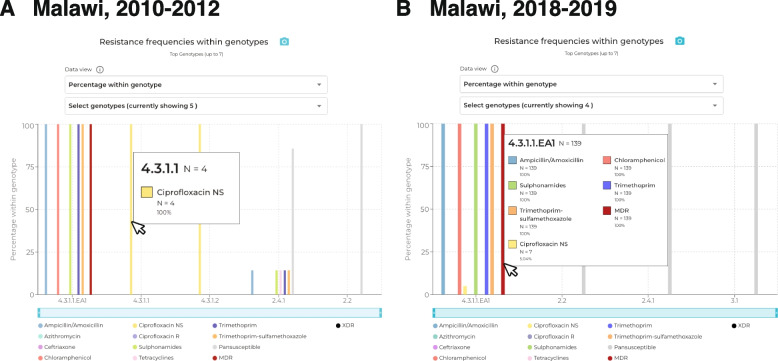
Genotypes associated with Ciprofloxacin non-susceptibility in Malawi in different periods. **A** In 2010–2012, CipNS was detected in *n* = 4 isolates of genotype 4.3.1.1 and *n* = 2 isolates of 4.3.1.2, which were susceptible to other drugs. **B** In 2018–2019, CipNS emerged in the MDR genotype 4.3.1.1.EA1 (*n* = 7 isolates)

The ‘Drug resistance trends’ plot from 2010–2019 (Additional file 1: Fig. S6b) demonstrated that CipNS was relatively low throughout this time period (< 16%; yellow line). However, there are two distinct periods within this time frame in which CipNS strains are observed; 2010–2012 and 2018–2019. By adjusting the start and end years to 2010 and 2012, respectively, using the dropdown menus in the top panel, it is apparent from the ‘Resistance frequencies within genotypes’ plot that CipNS during this early period was due to sporadic infections with CipNS genotypes 4.3.1.1 (*n* = 4) and 4.3.1.2 (*n* = 2) (Fig. 5a, Additional file 1: Fig. S6e). The ‘Resistance determinants within genotypes’ plot (with ‘Select drug class’ set to ‘Ciprofloxacin’, and ‘Data view’ set to ‘Number of genomes’) shows that genomes from both these genotypes carry one QRDR mutation (Additional file 1: Fig. S6f). However, when viewing the later period of CipNS strains (by adjusting start and end years to 2018 and 2019, respectively) the same two plots reveal that more recent CipNS cases are driven by the emergence of 1–2 QRDR mutations in the locally dominant genotype 4.3.1.1.EA1 (Fig. 5b, Additional file 1: Figs. S6 g-h). Strains that have acquired two QRDR mutations are of particular concern in this setting due to further elevating ciprofloxacin MIC, which is associated with increases in both fever clearance times and risk of clinical failures [29, 50, 61, 73].

The TyphiNET dashboard highlights the problem of MDR typhoid in Malawi, consistent with several recent genetic and phylogenetic studies [55, 74, 75]. These include (i) the time of MDR typhoid emergence; (ii) that MDR typhoid is associated with clonal replacement of local genotypes by 4.3.1.1.EA1, which now causes the majority of infections in this setting [55, 74]; and (iii) that CipNS is emerging in this setting, driven by the evolution of QRDR mutations in the now-endemic 4.3.1.EA1 strains [75]. Malawi has recently launched a national TCV immunisation program, motivated in large part by the sustained resistance to first-line drugs, and it will be important to monitor the impact of this on the pathogen population. Given the clinical relevance of ciprofloxacin, it will be particularly important to monitor resistance in Malawi and neighbouring countries, which will be facilitated by local and international sequencing efforts and data aggregation in TyphiNET.

### Limitations and future directions

The case studies presented here highlight how TyphiNET can be used to explore the Typhi populations in certain countries, capturing key aspects about the emergence and spread of AMR variants that are supported by current literature. However, the platform is necessarily limited by the availability of source data. India, Pakistan, Bangladesh, Nepal, and Malawi are among the countries with the most Typhi genome data available (*n* = 2327, *n* = 1526, *n* = 1664, *n* = 1300, *n* = 568, respectively), however, this is mainly from a handful of research studies [55, 56, 60, 63, 74, 75] that may not be representative of typhoid fever in each country. Indeed, one use of the dashboard is to highlight data gaps, which may help to prioritise areas for new typhoid surveillance. Routine sequencing of travel-associated cases in the UK and US contributed appreciable data for Pakistan (*n* = 32–217 per year) and Bangladesh (*n* = 15–47 per year), but none from Malawi and very few from Africa in general [23, 24]. The importance of pathogen sequencing in supporting infectious disease surveillance and public health response is increasingly recognised, including launch of the WHO-supported International Pathogen Surveillance Network (IPSN) [76] and the 2022–2032 WHO global genomic surveillance strategy for pathogens with pandemic and epidemic potential [77]. Regional and national efforts to strengthen WGS capacity in typhoid-endemic countries include Africa CDC’s Pathogen Genomics Initiative, regional PulseNet networks, and inclusion of WGS in national AMR surveillance in countries such as Pakistan, India, and the Philippines [78–80]. It can therefore be anticipated that the rate of Typhi sequencing is likely to increase, and the representation of cases from endemic areas will improve over time. However, the future utility of TyphiNET and other data aggregation efforts will depend on (i) the WGS data being shared, in a timeframe that is useful to inform and guide decision-making; (ii) the WGS data being accompanied by sufficient and accurate contextual metadata to be useful for epidemiological purposes; (iii) continuous curation of the AMR database hosted at Pathogenwatch; and (iv) ongoing development in response to stakeholder and community feedback. To support these needs, the GTGC was formed in 2021 to support and standardise generation, analysis and sharing of Typhi genome data. A contextual metadata template was developed that includes fields to capture the purpose of sampling as well as country of origin for travel-associated cases, and this has been used by the Consortium to curate public genome data for Pathogenwatch and TyphiNET. However, the ongoing relevance of public genome data, and downstream tools that utilise it such as TyphiNET, will depend on metadata standards and data sharing practices being adopted widely by those generating pathogen genomic surveillance data. We hope that this first version of TyphiNET illustrates a potential benefit of such data sharing, that might help encourage such practices. In the meantime, planned future developments to the dashboard include the addition of uncertainty measures (in addition to current visuals, which facilitate understanding limitations of the data by showing the sample size and count data for all data points plotted), and planned activities of the GTGC include aggregation, curation and linkage of AST data to support the ongoing accuracy of AMR predictions from phenotype, as new mechanisms and determinants emerge. Stakeholder engagement and feature development of TyphiNET, as well as extension to other typhoidal and non-typhoidal pathogens, is also ongoing with funding support under the AMRnet project [81]. 

## Conclusions

Against a backdrop of increasing AMR and vaccine rollouts in multiple countries, the TyphiNET dashboard provides an online resource for monitoring global and national genome-derived trends in AMR and pathogen variants without bioinformatics expertise. These data are potentially informative for implementing and monitoring vaccination and empirical treatment policies for typhoid fever, as well as understanding local variant transmission, enabling improved targeting of costly WASH interventions. As more Typhi WGS data become publicly available and curated by the GTGC, the TyphiNET database will be updated to provide a contemporary overview of ongoing and emerging trends in the pathogen population. Finally, the GTGC and TyphiNET approach provides a useful model for making pathogen genome-derived data broadly accessible that could be applied to other priority pathogens.

## Supplementary Information

Additional file 1. Supplementary tables and figures. (pdf format).

Additional file 2. TyphiNET downloadable report (pdf format) of all data available from Pakistan.

Additional file 3. TyphiNET downloadable line list (csv format) of all genome-derived data presented in TyphiNET.

## Data Availability

The TyphiNET dashboard concerns aggregating, processing and visualising genome sequencing data that is already in the public domain. Individual sample accession numbers for the sequences in the European Nucleotide Archive (https://www.ebi.ac.uk/ena) are given in Additional file 3 [24, 53, 54] and are also available in the database download (CSV file) that can be obtained from the dashboard site (https://www.typhi.net).

## Abbreviations

AMR: Antimicrobial Resistance
API: Application Programming Interface
AST: Antimicrobial Susceptibility Testing
AziR: Azithromycin Resistant
BIGSdb: Bacterial Isolate Genome Sequence Database
CefR: Ceftriaxone resistant
cgMLST: Core genome multilocus sequence typing
CipNS: Ciprofloxacin Non-susceptible
CipR: Ciprofloxacin Resistant
DDBJ: DNA Data Bank of Japan
EMBL-EBI: European Molecular Biology Laboratory—European Bioinformatics Institute
ENA: European Nucleotide Archive
GLASS: Global Antimicrobial Resistance and Use Surveillance System
GNU-GPL: General User Licence/General Public Licence
GTGC: Global Typhoid Genomics Consortium
H58: Haplotype 58 (genotype 4.3.1)
INSDC: International Nucleotide Sequence Database Collaboration
LMIC: Low- to Middle-Income Country
MDR: Multidrug Resistant
MERN: MongoDB, Express, React, Node
MIC: Minimal Inhibitory Concentration
NCBI: National Center for Biotechnology Information
NITAG: National Immunisation Technical Advisory Groups
PNG: Portable Network Graphics
PDF: Portable Documents Format
QRDR: Quinolone Resistance Determining Region
SEAP: Surveillance for Enteric Fever in Asia Project
STRATAA: Strategic Typhoid Alliance across Africa and Asia
SNP: Single Nucleotide Polymorphism
TCV: Typhoid Conjugate Vaccine
XDR: Extensively Drug Resistant
WGS: Whole Genome Sequencing
WHO: World Health Organization
